# When the tongue runs out of gas

**DOI:** 10.7554/eLife.86447

**Published:** 2023-02-28

**Authors:** Lila Wollman, Ralph Fregosi

**Affiliations:** 1 Department of Physiology, College of Medicine, University of Arizona Tucson Tucson United States

**Keywords:** sleep apnea, motoneuron, upper airway, hydrogen sulfide, carbon monoxide, Mouse

## Abstract

The transmission of signals from the brain to the tongue to control breathing depends, in part, on the balance between two gaseous molecules.

**Related research article** Browe BM, Peng YJ, Nanduri J, Prabhakar NR, Garcia AJ. 2023. Gasotransmitter modulation of hypoglossal motoneuron activity. *eLife* **12**:e81978. doi: 10.7554/eLife.81978.

Most of us spend little time thinking about our tongue, even though we depend on it for suckling, swallowing, chewing, speaking, tasting and other tasks. A lesser known but equally important role for the tongue is keeping the pharynx open so that breathing requires minimal effort, especially during sleep.

Every time we inhale, a region of the brain called the preBötzinger complex sends signals to hypoglossal motor neurons in the tongue. These neurons then activate the muscles in the tongue, causing them to contract so that the tongue remains forward in the mouth (Fregosi and Ludlow, 2014). However, if the tongue muscles do not activate fully, the tongue will move backwards, narrowing or even closing the pharynx.

Pharyngeal narrowing often occurs during sleep, because when we lie down – especially on our backs – gravity favors a backward movement of the tongue (Figure 1, top of central panel), requiring even more activity of the muscles to move the tongue forward (Marques et al., 2017). Moreover, the hypoglossal motor neurons become less active during sleep, which gives rise to snoring in susceptible people. While snoring is often harmless, it can be a precursor of the serious sleep-related breathing disorder known as obstructive sleep apnea (OSA).

**Figure 1.**
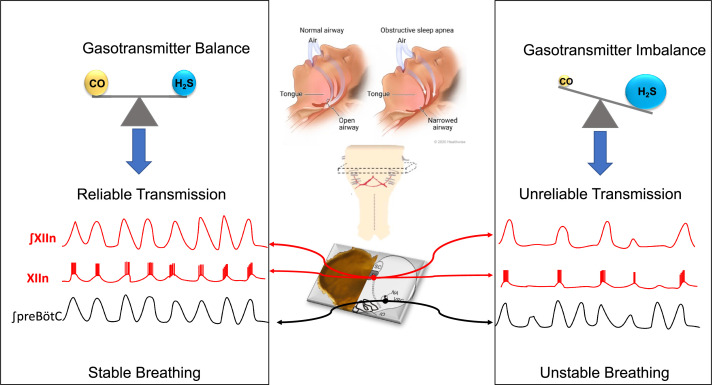
Gas signaling molecules and obstructive sleep apnea. When we sleep, the preBötzinger complex (a region of the brainstem) sends signals the hypoglossal motor neurons, activating the tongue muscles and causing them to contract: this means that the tongue stays forward to allow normal breathing (central panel, top left). In patients with obstructive sleep apnea, the tongue muscles are not sufficiently activated, so the tongue falls back and the pharynx becomes narrower, leading to snoring and difficulties breathing (central panel, top right). To study how the muscles of the tongue are activated by the preBötzinger complex when breathing, Browe et al. prepared ‘rhythmic brainstem slices’ by taking slices from the brainstem of mice (central panel, center) and recording their spontaneous respiratory activity (central panel, bottom). Browe et al. show that, in healthy mice (left panel), the balance of two gas signaling molecules, carbon monoxide (CO) and hydrogen sulfide (H_2_S), leads to the reliable transmission of the respiratory signal from the preBötzinger complex (signal shown in black) to the hypoglossal motor neurons (signals shown in red). When the activity of the HO-2 enzyme (not shown) is disrupted (right), the levels of CO decrease, leading to increased levels of H_2_S. This makes the signal transmission less reliable, which leads to an absence of activity in the hypoglossal motor neurons (red signals) during some respiratory cycles.

OSA is characterized by repeated episodes of pharyngeal narrowing or complete closure, and is associated with frequent episodes of labored breathing, followed by hypoxia (low levels of oxygen in the blood) and waking up. Unsurprisingly, these episodes are associated with loud snoring, fragmented sleep, and excessive drowsiness during the day (Abbasi et al., 2021). OSA also carries serious complications including increased risk of coronary artery disease, high blood pressure and type 2 diabetes.

It is estimated that between 5 and 15% of the general population in the United States has OSA (Lévy et al., 2015), and the incidence of OSA increases with obesity, likely because fat deposits on the wall of the pharynx cause it to narrow (Carter and Watenpaugh, 2008). The standard treatment for OSA is continuous positive airway pressure (CPAP), which involves wearing a special face mask connected to a machine that pressurizes the pharynx to widen it during sleep (Spicuzza and Sanna, 2021). Unfortunately, CPAP is uncomfortable and loud, and patients often do not use their CPAP machines consistently (Al-Abri et al., 2020). One way to address these issues is to identify the molecular mechanisms that control how the muscles in the tongue are activated, so drugs that promote this activation can be developed.

Now, in eLife, Alfredo Garcia III and colleagues at the University of Chicago – including Brigitte Browe as first author – report that the balance between two gases that act as signaling molecules help to regulate the activity of the neurons that activate the muscles of the tongue (Browe et al., 2023). Their work expands on previous research showing that mice lacking the enzyme heme oxygenase 2 (HO-2), which catalyzes the production of carbon monoxide, exhibit a higher incidence of OSA (Peng et al., 2017).

Carbon monoxide, a famously poisonous gas, is also an important signaling molecule in the brain. Browe et al. explored how perturbations in HO-2 activity that change carbon monoxide signaling can influence the activation of hypoglossal motor neurons (and potentially the muscles of the tongue). For their experiments, Browe et al. used a preparation of neural tissue known as the ‘rhythmic brainstem slice’. This technique allows researchers to study how signals are produced by pattern-generating neurons in the preBötzinger complex and then relayed through intermediate neurons to the hypoglossal motor neurons that project into the tongue muscles and activate them (Figure 1, bottom of central panel). Browe et al. first tested what would happen if the activity of the HO-2 enzyme was blocked in the rhythmic brainstem slice, either by inhibiting the enzyme pharmacologically or by deleting it through genetic manipulation. In both cases, blocking the activity of HO-2 – which presumably lowers carbon monoxide in the preparation – reduced the ability of the pattern-generating neurons to activate the hypoglossal motor neurons.

Next, Browe et al. showed that hydrogen sulfide, a gas produced by an enzyme regulated by carbon monoxide, can also reduce this signal transmission. When carbon monoxide and hydrogen sulfide are in balance, transmission of the signal from the preBötzinger complex to the hypoglossal motor neurons is reliable (Figure 1, left). In contrast, when the concentration of hydrogen sulfide exceeds that of carbon monoxide, transmission of the signal is less reliable, which can lead to breathing instability due to pharyngeal narrowing (Figure 1, right).

Together, the findings of Browe et al. indicate that carbon monoxide and hydrogen sulfide have opposing effects on the excitability of hypoglossal motor neurons, so balancing these two signaling molecules is likely to be important for regulating hypoglossal motor neuron activity and controlling the tongue’s position during breathing.
